# A Fast and Efficient Approach for Genomic Selection with High-Density Markers

**DOI:** 10.1534/g3.112.003822

**Published:** 2012-10-01

**Authors:** Vitara Pungpapong, William M. Muir, Xianran Li, Dabao Zhang, Min Zhang

**Affiliations:** * Department of Statistics, Purdue University, West Lafayette, Indiana 47907; † Department of Statistics, Chulalongkorn Business School, Bangkok, Thailand 10330; ‡ Department of Animal Science, Purdue University, West Lafayette, Indiana 47907; § Department of Agronomy, Kansas State University, Manhattan, Kansas 66506, and; ** School of Biomedical Engineering, Capital Medical University, Beijing, China 100069

**Keywords:** genotypic estimate of breeding values (GEBV), genomic selection, penalized orthogonal-components regression (POCRE), phenotypic estimate of breeding values (PEBV), GenPred, Shared data resources

## Abstract

Recent advances in high-throughput genotyping have motivated genomic selection using high-density markers. However, an increasingly large number of markers brings up both statistical and computational issues and makes it difficult to estimate the breeding values. We propose to apply the penalized orthogonal-components regression (POCRE) method to estimate breeding values. As a supervised dimension reduction method, POCRE sequentially constructs linear combinations of markers, *i.e.* orthogonal components, such that these components are most closely correlated to the phenotype. Such a dimension reduction is able to group highly correlated predictors and allows for collinear or nearly collinear markers. Different from BayesB, which predetermines hyperparameters, POCRE uses an empirical Bayes thresholding method to obtain data-driven optimal hyperparameters and effectively select important markers when constructing each component. Demonstrated through simulation studies, POCRE greatly reduces the computing time compared with BayesB. On the other hand, unlike fBayesB which slightly sacrifices prediction accuracy for fast computation, POCRE provides similar or even better accuracy of predicting breeding values than BayesB in both simulation studies and real data analyses.

The advantages of using genome-wide genetic markers to estimate the breeding values have been widely recognized (Meuwissen *et al.* 2001; Calus and Veerkamp 2007). Recent developments in high-throughput genotyping technology make it feasible and may further popularize the genomic selection in both plant and animal breeding programs (Meuwissen 2007; Zhong *et al.* 2009). However, such genomic selection practice is statistically challenging by analyzing a large number of genetic markers and efficiently estimating the breeding values. Moreover, high correlation between genetic markers and a relatively small number of individuals makes it difficult to directly employ classical statistical methods to estimate the breeding values.

A variety of methods have been proposed to estimate the total breeding value, for example, least squares (LS) approach, best linear unbiased prediction (BLUP), and Bayesian methods (Meuwissen *et al.* 2001, 2009). With a large number of genetic markers, the least squares approach relies on a suboptimal forward stepwise procedure to select significant genetic markers, and then uses them to predict the genetic values. The BLUP method is usually practiced using a single variance parameter to model the variation of all genes. While BLUP may be extended to allow for heterogeneous variances across loci, Meuwissen *et al.* (2001) proposed Bayesian models, which allow for heterogeneous variation across genes, and implemented BayesB using a Markov chain Monte Carlo algorithm. Unlike BLUP, which assumes genetic effects drawn from a normal distribution, BayesB implies a Student t-distribution that has heavier tails than the normal distribution and therefore can capture extreme genetic effects. To address the intensive computation required by BayesB, Meuwissen *et al.* (2009) employed an iterative conditional expectation (ICE) method to speed up the algorithm, hence named fBayesB, but slightly killed the accuracy of predictions.

Long *et al.* (2007) proposed a two-step machine learning classification procedure to select SNPs for genomic selection with binary traits. As a preprocessing method, an information gain-based (Mitchell 1997) filter was employed as the first step to select a small arbitrary number of genetic markers out of a huge number of candidates. At the second step, a naive Bayesian classifier (Elkan 1997) was adopted to optimize performance of the selected genetic markers. Although losing efficiency, dichotomizing phenotypic values was suggested to apply the above procedure for genomic selection with continuous traits (Long *et al.* 2007). Solberg *et al.* (2009) applied dimension reduction methods, such as the partial least squares regression (PLSR) and principal component regression (PCR) to genomic selection and showed that their performance was inferior to BayesB, although both methods were computationally faster and simpler.

The fact that an *L*_1_ penalty function can enable variable selection has recently motivated many penalized least squares methods to fit high-dimensional linear regression models, such as lasso proposed by Tibshirani (1996). Usai *et al.* (2009) applied the lasso method to estimate marker effects for genomic selection and concluded that it provided more accurate estimates of breeding values than BLUP and BayesA (Meuwissen *et al.* 2001). However, lasso lacks a grouping property, that is, it tends to select one marker from a group of highly correlated markers (Zou and Hastie 2005). Such a grouping property may play an important role in genomic selection, as markers from the same quantitative trait locus or genetic region are usually highly correlated and therefore may be preferred to be simultaneously included in or excluded from the prediction model. Many lasso variants have been proposed to take advantage of the grouped markers either implicitly or explicitly, for example, Zou and Hastie (2005), Tibshirani *et al.* (2005), and Yuan and Lin (2006).

Here we propose to apply the penalized orthogonal-components regression (POCRE) to estimate/predict breeding values and therefore enforce genomic selection. POCRE was proposed by Zhang *et al.* (2009) to select variables from high-dimensional, low-sample-size data. POCRE takes advantage of both Bayesian inference and supervised dimension reduction. With supervised dimension reduction, POCRE first constructs properly penalized orthogonal components, which are, upon standardization, most closely correlated to the response variables. A new penalization framework, implemented via empirical Bayes thresholding, is presented to effectively identify sparse markers for each component. POCRE is computationally efficient owing to its sequential construction of sparse orthogonal components. In addition, such construction offers other properties such as grouping highly correlated markers and allowing for collinear or nearly collinear markers.

On the basis of simulation studies, Meuwissen *et al.* (2001) compared four methods, including LS, BLUP, BayesA, and BayesB, and concluded that BayesB had the best overall performance. Therefore, we demonstrated the utility of POCRE in genomic selection by comparing it with BayesB using similar extensive simulation studies, and the performance was evaluated in terms of the prediction accuracy and computation time. We also applied both BayesB and POCRE to a pine dataset and a maize dataset to estimate the genetic values. With the data split into training subsets and test subsets, we compared the estimation accuracy.

## Methods

### Penalized orthogonal-components regression (POCRE)

With *p* markers genotyped from a total of *n* individuals, the classical regression model can be written as

(1) Y=μ*Xβ*ε

where **Y***_n_*_×1_ collects the phenotypic value of all individuals, **X***_n_*_×_*_p_* represents the genotype information of all individuals, *μ* is an overall mean of polygenic effects, *β_p_*_×1_ corresponds to the effect of each marker, and ε is the error term. Because typical genomic selection presents many more markers than individuals, in other words, *p* ≫ *n*, special algorithms are needed to estimate *β*. Zhang *et al.* (2009) recently described a penalized orthogonal-components regression (POCRE) approach that sequentially constructs sparsely loaded orthogonal components with proper regularization. They demonstrated that this approach works well when fitting regression models with *p* ≫ *n* data.

Assume that both **Y** and **X** are centralized, and accordingly, assume *μ* = 0 in Model 1. POCRE sequentially constructs orthogonal components ${\tilde{X}}_{1}\omega_{1}$, ${\tilde{X}}_{2}\omega_{2}$, …, where ${\tilde{X}}_{1}=X$ and ${\tilde{X}}_{k}$, *k* ≥ 2, are iteratively built to be orthogonal to $\left\{{\tilde{X}}_{1}\omega_{1},\ldots ,{\tilde{X}}_{k-1}\omega_{k-1}\right\}$. The loading *ω_k_*, *k* ≥ 1, is obtained as γ/‖γ‖ which together with *α* minimizes

(2) $${\left\|\gamma -{\tilde{X}}_{k}^{T}YY^{T}{\tilde{X}}_{k}\alpha \right\|}^{2}*g_{\lambda }\left(\gamma \right),\;\;\text{subject to}\left\|\alpha \right\|=1,$$

where *g_λ_*(*γ*) is a penalty function defined by a proper regularization on *γ* with tuning parameter *λ*.

When *g_λ_*(*γ*) ≡ 0, the optimal *γ* solving Equation 2 is proportional to the leading eigenvector of ${\tilde{X}}_{k}^{T}YY^{T}{\tilde{X}}_{k}$, that is, $\omega_{k}={\tilde{X}}_{k}^{T}Y/\left\|{\tilde{X}}_{k}^{T}Y\right\|$ when a single phenotype is considered. Zhang *et al.* (2009) employed the empirical Bayes thresholding method proposed by Johnstone and Silverman (2004) to introduce proper penalty *g_λ_*(*γ*). While POCRE_0_ directly applies the empirical Bayes thresholding method, POCRE applies it to the statistics from Fisher’s z-transformation. Such penalty helps estimating covariance between phenotype and genotypes, and it provides adaptively sparse loadings of orthogonal components. The empirical Bayes implementation is also computationally efficient. The tuning parameter *λ* can be appropriately set to account for possible dependence structure among different markers.

The sequential construction of the orthogonal components stops when the optimal *γ* solving Equation 2 is zero, which implies that $\tilde{X}$ is almost uncorrelated to **Y**. Then regressing **Y** on the orthogonal components, *i.e.* ${\tilde{X}}_{1}\omega_{1}$, ${\tilde{X}}_{2}\omega_{2}$, …, provides estimates of *β*_1_,…, *β_p_* in Model 1. Since non-zero loadings in *ω_j_*, *j* = 1, 2,…, are sparse, most of the estimated *β*_1_,…, *β_p_* are therefore zero, reflecting the fact that most markers do not (significantly) contribute to predicting phenotypic values, as discussed in the literature, *e.g.* Hayes *et al.* (2009). Note that, when the above sparsity argument is violated, it is preferable to set *g_λ_*(*γ*) ≡ 0 in Equation 2, which essentially leads to the partial least squares algorithm (Wold 1975) by selecting an optimal number of components.

### Choice of the tuning parameter *λ*

While the tuning parameter *λ* is used to account for possible dependence structure among different markers, it can be chosen on the basis of previous experience with similar studies. In the case of no experience with similar studies, a simulation study may be designed to utilize the available genotypic values and to reveal an appropriate choice of the tuning parameter. We will discuss several strategies on the basis of cross-validation (CV) to elicit a data-driven optimal value for the tuning parameter in the simulation studies.

### Simulation studies

A gene-level simulation program was utilized as described by Muir (2007). For this set of simulations, the conditions and assumptions were as similar to Meuwissen *et al.* (2001) as possible. Specifically, the genetic architecture included 1000 SNP loci and 1000 QTL, both biallelic. There were a total of 10 chromosomes, and the length of each chromosome was 100 cM, which implies one marker per centimorgan. The 1000 SNP loci were equally spaced, whereas the 1000 QTL were randomly generated from a uniform distribution across the genome. The allelic effects were additive and drawn from a gamma distribution with a shape parameter of 0.4 and scale parameter of 1.66, meaning there will be few alleles with large effects. The mutation rate was set at 2.5 × 10^−5^ and 2.5 × 10^−3^ for QTL and marker SNP, respectively, per locus per generation. On the basis of the genetic variance, environmental effects were generated from a Gaussian distribution with mean zero and variance calculated to meet the required heritability, which is 0.5 in the simulation. To establish mutation drift equilibrium, a population of 100 (50 males and 50 females) were allowed to randomly mate for 1000 generations. Each family produced 2 offspring. The population was expanded by twice in the next generation (*i.e.* 200 individuals at generation 1001) and then by a factor of 10 in the following generations (*i.e.* 2000 individuals for each generation after generation 1001). The 2200 individuals at generations 1001 and 1002 were genotyped and phenotyped, respectively (Muir 2007), and served as the training data. The remaining generations, serving as the test data, were only genotyped but not phenotyped, although the true breeding values were generated for testing the accuracy of models. A total of 50 replicates were simulated.

As shown above, genomic selection can proceed on a population consisting of several generations. For instance, the data in simulation studies include two generations: 200 individuals from generation 1001, and 2000 individuals from generation 1002. With different ways to split the data, we consider three implementations of 10-fold CV in analyzing these datasets. The first strategy, termed as CV0, is to ignore the mixed generations and to randomly split 2200 individuals in each sample into 10 different folds, that is, each with 220 individuals. The second strategy, termed as CV1, always includes all 200 individuals from generation 1001 in the training data and only splits 2000 individuals from generation 1002 into 10 different folds. Therefore, each test data will only include 200 individuals from generation 1002 but none from generation 1001. The third strategy, termed as CV2, separately splits the 200 individuals from generations 1001 and the 2000 individuals from generation 1002 into 10 folds, such that each fold includes 20 individuals from generation 1001 and 200 individuals from generation 1002.

POCRE and POCRE_0_ were employed to analyze all simulated datasets with the tuning parameter elicited via cross-validation. All three cross-validation techniques (CV0, CV1, and CV2) were used in the analyses. Here we applied BayesB and compared it with POCRE and POCRE_0_ in terms of the computation time and the prediction accuracy, which is defined as the correlation between the estimated and true breeding values. For BayesB, the probability *π* that a SNP has zero effect is assessed empirically from the data, and the other hyperparameters were chosen following Meuwissen *et al.* (2001).

## Results

### Prediction accuracy

A striking nature of BayesB is that it employs a mixture prior for each regression coefficient in Equation 1 and allows it to take zero value with positive probability. Each coefficient in BayesB was estimated from the posterior mean, and a property of this estimator is that all means are non-zero. Though fBayesB has computational advantages over BayesB, Meuwissen *et al.* (2009) demonstrated that fBayesB is slightly less accurate than BayesB in terms of correlation between estimated breeding values (EBV) and true breeding values (TBV). Throughout this article, we shall focus on comparing accuracy between POCRE and BayesB only.

As shown in Table 1, all POCRE and POCRE_0_ methods provided better estimates of breeding values for generations 1001 and 1002 than BayesB. For POCRE and POCRE_0_ methods, their predictions of breeding values for generation 1003 were not only more accurate but also more robust than those of BayesB. Indeed, except for generation 1004 with POCRE_0_ (CV0), all POCRE implementations provided more robust predictions of breeding values than BayesB.

**Table 1:** Prediction accuracy of BayesB and POCRE

	Generation					
Method	1001/1002	1003	1004	1005	1006	1007
BayesB	0.7038	0.5008	0.4636	0.3716	0.3670	0.3096
	(0.0399)	(0.0460)	(0.0423)	(0.0468)	(0.0426)	(0.0428)
POCRE(CV0)	**0.7392**	**0.5138**	0.4503	0.3651	0.3490	0.2983
	(0.0407)	(**0.0419**)	(**0.0394**)	(**0.0402**)	(**0.0375**)	(**0.0392**)
POCRE(CV1)	**0.7396**	**0.5186**	0.4573	0.3676	0.3563	0.3086
	(**0.0369**)	(**0.0399**)	(**0.0369**)	(**0.0396**)	(**0.0385**)	(**0.0399**)
POCRE(CV2)	**0.7413**	**0.5170**	0.4526	0.3673	0.3535	0.3066
	(0.0408)	(**0.0427**)	(**0.0382**)	(**0.0395**)	(**0.0341**)	(**0.0376**)
POCRE_0_(CV0)	**0.7360**	**0.5124**	0.4430	0.3654	0.3482	0.2974
	(0.0456)	(**0.0427**)	(0.0430)	(**0.0445**)	(**0.0387**)	(**0.0404**)
POCRE_0_(CV1)	**0.7351**	**0.5142**	0.4532	0.3715	0.3561	0.3074
	(**0.0383**)	(**0.0362**)	(**0.0363**)	(**0.0377**)	(**0.0347**)	(**0.0351**)
POCRE_0_(CV2)	**0.7425**	**0.5197**	0.4541	**0.3732**	0.3493	0.3061
	(0.0427)	(**0.0388**)	(**0.0401**)	(**0.0412**)	(**0.0385**)	(**0.0393**)

Estimated *corr*(*TBV*, *EBV*) and corresponding standard deviations (in parentheses) among 50 simulated datasets. Values shown in bold indicate better performance than BayesB.

When comparing the three different strategies of cross-validation for both POCRE and POCRE_0_, we observe that CV0 was the worst strategy as it always provided the lowest accuracy in predicting breeding values among all three strategies for both POCRE and POCRE_0_. For POCRE, CV1 performed the best by providing better prediction accuracy than the other two. However, for POCRE_0_, CV2 provided the best prediction accuracy in generations 1003–1005, whereas CV1 provided the best prediction accuracy in generations 1006 and 1007. In general, prediction accuracy between CV1 and CV2 was only slightly different for both POCRE and POCRE_0_. All cross-validation strategies were similarly robust in predicting breeding values. Therefore, we suggest using either CV1 or CV2 scheme for cross-validation to choose the tuning parameters for both POCRE and POCRE_0_.

It is well known that the coefficient in a non-intercept simple regression of TBV against EBV, denoted as *β*(*TBV*, *EBV*), can be used to evaluate the bias of genomic selection methods. With *β*(*TBV*, *EBV*) = 1, the method provides unbiased prediction of the breeding values; *β*(*TBV*, *EBV*) < 1 implies an overestimated breeding value, and *β*(*TBV*, *EBV*) > 1 implies an underestimated breeding value. Table 2 shows the estimated *β*(*TBV*, *EBV*) of all methods across different generations. The predictions of all methods alternated between overestimate and underestimate from generations 1003 to 1007. That is, these methods all overestimated/overpredicted the breeding values in generations 1001/1002, 1004, and 1006, but they underpredicted the breeding values in generations 1003, 1005, and 1007. In general, all methods performed quite well in predicting the breeding values in terms of bias.

**Table 2:** Comparison of the estimated *β*(*TBV*, *EBV*) using BayesB and POCRE

	Generation					
Method	1001/1002	1003	1004	1005	1006	1007
BayesB	0.99999	1.00015	0.99998	1.00015	0.99999	1.00010
POCRE(CV0)	0.99997	1.00020	1.00000	1.00020	0.99996	1.00010
POCRE(CV1)	0.99997	1.00020	0.99997	1.00020	0.99994	1.00020
POCRE(CV2)	0.99997	1.00020	0.99998	1.00020	0.99995	1.00010
POCRE_0_(CV0)	0.99996	1.00020	0.99992	1.00020	0.99994	1.00015
POCRE_0_(CV1)	0.99997	1.00010	0.99996	1.00015	0.99995	1.00010
POCRE_0_(CV2)	0.99997	1.00020	0.99996	1.00015	0.99994	1.00020

Total of 50 simulated datasets.

### Computing time

As one of the primary goals of this study was to propose a fast approach for genomic selection, we compared the computing time of BayesB with POCRE. All methods were programmed in MATLAB, and all simulated data were analyzed on a Linux server with dual Intel Xeon Quad-Core 5410 2.33 GHz processors without taking advantage of parallel computation. We witnessed that the computing times for POCRE and POCRE_0_ were similar and both took much less time than BayesB. Specifically, BayesB took 14,685.12 sec for one dataset based on 5,000 burn-ins and another 5000 iterations for inference.

When the tuning parameters of POCRE and POCRE_0_ need to be elicited through cross-validation, the computation time relies on the number of folds for cross-validation and the number of candidate values searched for the tuning parameter. For example, when a 10-fold cross-validation is utilized to search the tuning parameter from the pool {0.7, 0.71,…, 0.8}, 101 datasets need to be analyzed by either POCRE or POCRE_0_ for choosing the tuning parameter; therefore, it took about 1010 sec to search for the optimal tuning parameter and another 10 sec to fit the model. Hence, the computation time is about 1/14 of that of BayesB. To further reduce the computation time of POCRE and POCRE_0_, some strategies (discussed later) can be designed to search for a much smaller number of candidate tuning parameter values during cross-validation, and the number of folds can be reduced when a large number of individuals is available. Indeed, available multiple processors in many computers imply that parallel computation can greatly shorten the computing time of POCRE and POCRE_0_.

### Real data analyses

Although genomic selection emerged from dairy cattle breeding programs, it is also useful to plant breeding programs (Jannink *et al.* 2010; Bernardo and Yu 2007). To further demonstrate the utility of the proposed method, we applied the methods to two datasets: pine and maize.

#### Pine data:

The pine dataset consists of 850 individuals and 4698 SNPs. Missing genotypes were imputed by sampling alleles from a Bernoulli distribution with variance equal to locus allele frequency. Individuals and SNPs having more than 20% missing values were removed from the data before the analysis. Diameter at breast height (DBH) and height (HT) were collected for pine trees in Nassau that were used in our analysis. Details of this data are referred to in Resende *et al.* (2012).

Here we applied POCRE and POCRE_0_ to evaluate their performance with 10-fold cross-validation because independent validation population is not available. As shown in Table 3, POCRE and POCRE_0_ yielded similar accuracies. Indeed, POCRE provided more robust accuracies for both DBH and HT traits. Accuracies of estimated breeding values for POCRE were also better than POCRE_0_, except in the training data of HT trait. The results of POCRE and POCRE_0_ were similar in terms of the bias as shown in Table 4.

**Table 3:** Results of POCRE and POCRE_0_ for analyzing pine dataset

	Trait			
	DBH		HT	
Method	Training	Test	Training	Test
POCRE	**0.9669**	**0.6943**	0.9636	**0.6413**
	(**0.0025**)	(**0.0450**)	(**0.0017**)	(**0.0526**)
POCRE_0_	0.9661	0.6900	**0.9640**	0.6366
	(0.0029)	(0.0472)	(0.0023)	(0.0550)

Estimated correlation coefficients between the observed and estimated phenotypic values and the corresponding standard deviations (in parentheses) based on 10-fold random cross-validation. Values in bold indicate the better performance in each column.

**Table 4:** Results of POCRE and POCRE_0_ for analyzing pine dataset

	Trait			
	DBH		HT	
Method	Training	Test	Training	Test
POCRE	0.8999	0.7444	0.9181	**0.7393**
	(**0.0181**)	(**0.0585**)	(**0.0125**)	(**0.0440**)
POCRE_0_	**0.9067**	**0.7472**	**0.9200**	0.7349
	(0.0198)	(0.0612)	(0.0137)	(0.0444)

Estimated *β*(*TBV*, *EBV*) and corresponding standard deviations (in parentheses) based on 10-fold random cross-validation. Values in bold indicate the better performance in each column.

#### Maize data:

This data were collected from a maize study on flowering time (Buckler *et al.* 2009). The maize nested association mapping (NAM) population, consisting of 5000 recombinant inbred lines (RIL), was created by crossing 25 diverse lines to a common parent, B73 (Yu *et al.* 2008). The phenotype data for days to anthesis (DA, male flowering) collected for 5000 RILs were used in this analysis; this trait has an estimated heritability around 94% (Buckler *et al.* 2009). The genotype data are SNPs with 5% or more minor allele frequency across 10 chromosomes, a total of 284,025 SNPs. Briefly, all 5000 NAM RILs and 26 parents were genotyped with 1106 B73-specific tag SNPs (tSNP) across the genome, and additional SNPs were obtained through sequencing of the 26 parents (Gore *et al.* 2009). Imputations were performed with fastPHASE (Scheet and Stephens 2006) to fill the data if a SNP was missing in less than 5 parents, and the resulting SNP data were then projected to 5000 RILs based on tSNPs (McMullen *et al.* 2009; Yu *et al.* 2008).

We randomly split the data into training and test sets of sizes 3500 and 1392, respectively, to evaluate the performance of our method. Five replicates were carried out to test the accuracy, which results in five pairs of training and test sets. Without the true breeding values (TBV), we can estimate them using either the original phenotypic values (termed PEBV hereafter) or genotypic values (termed GEBV hereafter) with BayesB or POCRE. The correlation between PEBV and GEBV, that is, *corr*(*PEBV*, *GEBV*), was estimated to assess the accuracy of genomic selection. Indeed, $corr\left(PEBV,GEBV\right)=corr\left(TBV,GEBV\right)/\sqrt{h^{2}}$ with *h*^2^ referring to the heritability.

With the large number of SNPs included in this example, it was computationally intensive to run the Markov chain Monte Carlo for BayesB. Therefore, we only applied POCRE and POCRE_0_ to analyze the whole-genome dataset, with 5-fold cross-validation to elicit the tuning parameters. Table 5 shows the results based on the five replicates. For the training datasets, we observed that POCRE provided estimated *corr*(*PEBV*, *GEBV*) averaged at 0.9381, and POCRE_0_ at 0.9426; both are very close to the estimated heritability 94%. Both methods are robust in estimating the breeding values of the training datasets. For the test datasets, both methods provided robust estimates of breeding values, with estimated standard deviations 0.0045 and 0.0039 for POCRE and POCRE_0_, respectively, without the first replicate. Table 6 shows that the bias of the estimation is minimal.

**Table 5:** Results of analyzing maize flowering time data using POCRE and POCRE_0_

		POCRE		POCRE_0_	
		Training Data	Test Data	Training Data	Test Data
	1	0.9348	0.6763	0.9406	0.6988
	2	0.9437	0.9096	0.9483	0.9140
Replicate	3	0.9268	0.8992	0.9399	0.9072
	4	0.9418	0.9060	0.9382	0.9066
	5	0.9433	0.9076	0.9460	0.9053
MEAN		0.9381	0.8597	0.9426	0.8664
STDEV		0.0073	0.1026	0.0043	0.0937

Estimated correlation coefficients between PEBV and GEBV, *i.e. corr*(*PEBV*, *GEBV*).

**Table 6:** Results of the estimated *β*(*TBV*, *EBV*) in analyzing maize flowering time data using POCRE and POCRE_0_

		POCRE		POCRE_0_	
		Training Data	Test Data	Training Data	Test Data
	1	1.0001	0.9783	1.0000	0.9782
	2	1.0000	1.0002	1.0001	0.9999
Replicate	3	0.9998	1.0002	1.0001	1.0002
	4	0.9999	1.0004	0.9999	1.0004
	5	1.0000	0.9995	1.0000	0.9995
MEAN		0.9999	0.9957	1.0000	0.9956
STDEV		0.0001	0.0097	0.0001	0.0097

To compare BayesB, POCRE, and POCRE_0_, we chose 2000 SNPs based on univariate tests performed for each SNP separately. From the SNPs with *P*-values less than 0.0001, we randomly selected 200 SNPs, and we chose 1800 SNPs from those with *P*-values greater than 0.0001. With the selected 2000 SNPs, we applied all methods to re-analyze the five replicates. Five-fold cross-validation was employed to elicit the tuning parameter for POCRE and POCRE_0_. The results are summarized in Tables 7 and 8. Both POCRE and POCRE_0_ provided the larger estimate of *corr*(*PEBV*, *GEBV*) for the training datasets, and they predicted the breeding values with better accuracy and more robustly than BayesB. Note that with only 2000 SNPs, *corr*(*PEBV*, *GEBV*) estimated from the test datasets are smaller than those presented in Table 5.

**Table 7:** Comparison of BayesB and POCRE for analyzing the maize flowering time data using 2000 SNPs

Method	Training Data	Test Data
BayesB	0.8084 (0.0094)	0.6572 (0.3049)
POCRE	**0.9145** (**0.0078**)	**0.8131** (**0.1590**)
POCRE_0_	**0.9165** (**0.0054**)	**0.7741** (**0.2456**)

Estimated correlation coefficients between the observed and estimated phenotypic values and the corresponding standard deviations (in parentheses). Values in bold indicate better performance than BayesB.

**Table 8:** Comparison of BayesB and POCRE for analyzing the maize flowering time data using 2000 SNPs

Method	Training Data	Test Data
BayesB	1.0016 (0.0388)	1.0233 (0.0281)
POCRE	**0.9998** (**0.0001**)	**0.9951** (**0.0096**)
POCRE_0_	**1.0000** (**0.0001**)	**0.9954** (**0.0091**)

Estimated *β*(*TBV*, *EBV*) and the corresponding standard deviations (in parentheses). Values in bold indicate better performance than BayesB.

## Discussion

Here we present alternative approaches for genomic selection that provide competitive prediction of breeding values and also significantly reduce the computing time. First, note that the prediction accuracy of both POCRE and POCRE_0_ are comparable to that of BayesB in simulation studies, and their performance is much better than BayesB in analyzing maize flowering time data. Second, POCRE and POCRE_0_ enjoy enormous computational advantage over BayesB. Finally, POCRE and POCRE_0_ make it feasible to analyze data with a huge number of markers. For example, both POCRE and POCRE_0_ (with tuning parameters elicited via 5-fold cross-validation) took about 36 hr to analyze the maize flowering time data, which includes a total of 284,025 SNPs for each of the 5000 genetic lines. This is promising because current high-throughput biotechnologies routinely genotype millions of markers. Our proposed method has the potential to take advantage of such high-dimensional data that may challenge the genomic selection.

Results from both simulation studies and real data analysis showed the importance of the tuning parameter for the performance of POCRE and POCRE_0_. While cross-validation is a popular method to select the tuning parameter, it may not be optimum as shown in our simulation studies and others (Wang *et al.* 2007). Because the squared error loss was used as a criterion in cross-validation to select the optimal tuning parameter with training data, it suffers from overfitting the model, even with a large sample size. To explore various choices of tuning parameter, wider ranges can be used. Optimal tuning parameters in different genomic selection studies may differ considerably, and this issue is still under investigation.

As evidenced in the simulation studies, selecting the tuning parameter using cross-validation should also consider the possible population structure in the data. The time usage for cross-validation relies on the number of folds and number of potential tuning parameter values under investigation. Parallel computation can significantly reduce the computation time of cross-validation. As genomic selection datasets usually include a large number of individuals, we can choose the number of folds as small as two. We have considered a grid search of the optimal tuning parameter in the simulation studies and real data analysis. For a strategic thorough search, we may first take a coarse grid to search and then look into a finer interval suggested from the initial coarse grid search. On the other hand, some targeted simulation studies may be designed to shed light on the optimal tuning parameter for a specific study.

## Supplementary Material

Supporting Information
