# Supplementary material for: A Fast and Efficient Approach for Genomic Selection with High-Density Markers
Source: G3 (Bethesda). 2012 Oct 1;2(10):1179–84. doi: 10.1534/g3.112.003822 (PMC3464110; doi:10.1534/g3.112.003822)
Supplement: Supporting Information [file supp_2_10_1179__index.html]

Supporting Information 

# A Fast and Efficient Approach for Genomic Selection with High-Density Markers

## Supporting Information for Pungpapong *et al.*, 2012

**Files in this Data Supplement:**

- File S1 - Supporting Data (PDF, 189 MB)
